# Polymorphism and mutational diversity of virulence (*vcgCPI/vcgCPE*) and resistance determinants (*aac(3)-IIa, (aacC2*, *strA*, *Sul 1*, and *11*) among human pathogenic *Vibrio* species recovered from surface waters in South-Western districts of Uganda

**DOI:** 10.1186/s43141-023-00554-1

**Published:** 2023-10-06

**Authors:** Hope Onohuean, Uchechukwu U. Nwodo

**Affiliations:** 1 Biopharmaceutics Unit, Department of Pharmacology and Toxicology, School of Pharmacy, Kampala International University Western Campus, Ishaka-Bushenyi, Uganda; 2 Biomolecules, Metagenomics, Endocrine and Tropical Disease Research Group (BMETDREG), Kampala International University, Western Campus, Ishaka-Bushenyi, Uganda; 3 Patho‑Biocatalysis Group (PBG), Department of Biochemistry and Microbiology, University of Fort Hare, Private Bag 1314, Alice, 5700 Eastern Cape South Africa

**Keywords:** Polymorphism, Mutational diversity, Nucleotide sequence, Putative-amino-acids, Restriction enzymes, Virulence, Resistance genes, *Vibrio* species, Surface waters

## Abstract

**Background:**

*Vibrio* species are among the autochthonous bacterial  populations found in surface waters and associated with various life-threatening extraintestinal diseases, especially in human populations with underlying illnesses and wound infections. Presently, very diminutive information exists regarding these species’ mutational diversity of virulence and resistance genes. This study evaluated variations in endonucleases and mutational diversity of the virulence and resistance genes of *Vibrio* isolates, harboring virulence-correlated gene (*vcgCPI*), dihydropteroate synthase type 1 and type II genes (*Sul 1* and *11*), *(aadA)* aminoglycoside (3′′) (9) adenylyltransferase gene, *(aac(3)-IIa, (aacC2)a*, aminoglycoside N(3)-acetyltransferase III, and (*strA*) aminoglycoside 3′-phosphotransferase resistance genes.

**Methods:**

Using combinations of molecular biology techniques, bioinformatics tools, and sequence analysis.

**Results:**

Our result revealed various nucleotide variations in virulence determinants of *V. vulnificus* (*vcgCPI*) at nucleotide positions (codon) 73–75 (A → G) and 300–302 (N → S). The aminoglycosides resistance gene (*aadA*) of *Vibrio* species depicts a nucleotide difference at position 482 (A → G), while the aminoglycosides resistance gene (*sul 1* and *11*) showed two variable regions of nucleotide polymorphism (102 and 140). The amino acid differences exist with the nucleotide polymorphism at position 140 (A → E). The banding patterns produced by the restriction enzymes *HinP1I*, *MwoI*, and *StyD4I* showed significant variations. Also, the restriction enzyme digestion of protein dihydropteroate synthase type 1 and type II genes (*Sul 1* and *11*) differed significantly, while enzymes *DpnI* and *Hinf1* indicate no significant differences. The restriction enzyme *NlaIV* showed no band compared to reference isolates from the GenBank. However, the resistant determinants show significant point nucleotide mutation, which does not produce any amino acid change with diverse polymorphic regions, as revealed in the restriction digest profile.

**Conclusion:**

The described virulence and resistance determinants possess specific polymorphic locus relevant to pathogenomics studies, pharmacogenomic, and control of such water-associated strains.

**Supplementary Information:**

The online version contains supplementary material available at 10.1186/s43141-023-00554-1.

## Background

Most *Vibrio* species are human pathogens [1, 2] and disease-causing strains that have been particularly implicated in gastroenteritis and the infection of open wounds, causing sepsis [3]. These species are primarily present in water and food and carried by many marine animals, such as crabs or prawns, which carry the bacteria that can cause fatal illnesses if exposed [4–7].

Several genomic, proteomic, and genetic markers have been applied to the pathogenic profile of the water-loving *Vibrio* species [6]. In particular, primary pathogenic/epidemic genetic markers/genes for *V. cholerae* include *ctxAB*, *tcpA*, hap, and *toxR,* which codes for cholera toxin, toxin-coregulated adhesion pili, soluble hemagglutinin/protease, and regulatory toxoid [2, 8, 9]. While *V. parahaemolyticus* has the genetic marker O3:K6 antigens that regulate the serovar, also the genes *toxRS* [10], *orf8* [11], and *tdh*; and *trh*, found in most of the pathogenic strains. The *V. Vulnificus* markers involve pathogenicity region XII, nanA, and a mannitol fermentation operon containing alleles of the 16S rRNA and vcg genes linked with pathogenicity [12]. *V*. *mimicus* genetic factors include; quorum-sensing regulation system, hemolysins, proteases, outer membrane proteins [(*OmpU*), *OmpT*, *OmpK*, and *OmpV*] [2], a type IV and MSHA pilus, an aerobactin siderophore, a capsular polysaccharide, an accessory colonization factor (*acfD*), the transmembrane regulatory protein *ToxS*, the transcriptional activator *ToxR*, and the presence of quorum- (*LuxS*, *LuxO*, *LuxR*) [13]. While other pathogenic vibriosis shares common and/or combined genetic markers. It is imperative to note that some *Vibrio* spp., show no positive result to the aforementioned genetic markers but are potential pathogens, implying the discrimination markers insufficient to trace the toxins in the bacterial isolate in environment samples [7].

In addition, multiple drug resistance is well reported among the *Vibrio* strains highlighting mechanisms via resistance coding genes [9], the acquisition of conjugative plasmids [14–16], genetic elements (class 1 integron and *SXT* elements), a potential carrier of antimicrobial resistance genetic determinants [9, 17, 18]. Also, conjugative elements (ICEs) are a type of mobile genetic element that encodes various characteristics, including drug resistance [19]. Specifically, the SXT element helps horizontal resistance gene transfer and rearrange resistance genes in *V*. *cholerae*. It was initially found in the V. *cholerae* O139 MO10 chromosome from India (SXTMO10) but was later observed in other strains [20]. This element can mobilize plasmids, integron genes, and other resistant genes, including chloramphenicol (coded by floR), streptomycin (*strA* and *strB*), sulfamethoxazole (*sul1* and *sul2*), trimethoprim (*dfrA18*), Penicillins (*AmpC*), lactamase for Cephalosporins, (*blaSHV*, *blaTEM*, *blaCTX-M*) Carbapenems (*blaNDM-1*, *blaKPC*, *blaIMP, blaVIM*), Macrolides (*vanA, mecA*), and Fluoroquinolones (*mcr-1*) and tetracycline (*tetA* gene) [7, 21–24]. High levels of resistance to sulfamethoxazole (*sul2*), chloramphenicol (*floR*), streptomycin (*strA* and *strB*), and trimethoprim (*dfrA1*) have been documented [18], which are associated with the integrase gene, *SXT int*, and associated SXT resistance genes. At the same time, there are variant types of the *SXT* element among pathogenic *Vibrio* spp. (*Vibrio vulnificus*, *Vibrio metschnikovii*, *Vibrio fluvialis*, and *Vibrio parahaemolyticus*) harbor these resistance genes [2, 25, 26].

Understanding the wide variations or mutations in virulence and resistance genes, including genetic and pathogenic diversity in natural environments among *Vibrio* species, are important and relevant indices for control, especially among other strains of *Vibrio*. Like other infectious diseases, typically fluoroquinolone resistance has been attributed to amino acid changes at positions Ser79 of ParC and Ser81 of GyrA to either Phe or Tyr (8, 33) [27, 28]. However, the appropriate codons' single-base modifications cannot account for these amino acid alterations, often they are second-step substitutions caused by 2-bp changes to the serine codons at ParC (TCT to CTT) or GyrA (TCC to ATC), respectively [27]. Also, mutations detected in the QRDRs of GyrA (Ser83-Ile) and ParC (Ser85-Leu) revealed the mechanisms for nalidixic acid resistance among *Vibrio* strains [26]. These mutations of a set of mobile fluoroquinolone resistance genes (qnr-genes), are implicated in the contamination of microbial communities. For instance, the chromosomal resistance mutations can arise de novo and become abundant in a population with strong sufficient antibiotic selective pressure, thereby confirming clinically relevant resistance. However, the abundance and distributions of these chromosomal resistance mutations in environmental bacterial communities are poorly investigated.

Pulsed-field gel electrophoresis (PFGE) uses appropriate restriction enzymes to break down bacterial DNA at a select few locations in the genome, resulting in big or macro-DNA fragments that may be sorted based on size. It has been demonstrated that PFGE banding patterns produced by NotI restriction are a useful genotypic tool for identifying *V. cholerae* O1 strains [29]. Comparison of these restriction enzyme profiling could indicate whether isolates are epidemiologically linked to understanding regional diversity and global distribution for comprehensive ancestry analysis of pathogenic *Vibrio* spp. [30]. Therefore, this study assesses the polymorphism and mutational diversity of the nucleotide and putative amino acid sequences of virulence (*vcgCPI* and *vcgCPE*) and resistance determinants (*aac(3)-IIa*, *(aacC2*, *strA*, *Sul 1*, and *11*) found among human pathogenic *Vibrio* species that were recovered from surface waters in South-Western districts of Uganda.

## Methods

### Collection of samples, processing, and enumeration of Vibrio spp.

A total of 230 water samples were collected from 46 villages between June 2018 and October 2018. Using sterilized Nalgene glass bottles, (1000 ml) water samples were collected from different sampling points in each of the four districts (including, Bushenyi, Mitooma, Rubirizi, and Sheema) in South West of Uganda and transported in an ice-cool box to the laboratory for analysis within 6 h. tenfold dilutions were carried out on the water samples as described by Adefisoye and Okoh (2016) [31], 1 mL of each serial dilution was plated onto TCBS agar (thiosulphate citrate bile salts sucrose) (Neogen, Lansing, MI 48912 USA) in triplicates for 24 h and incubated at 37 °C. The presumptive *Vibrio* spp., was then counted and measured in colony-forming units per milliliters (CFU/mL) of water samples for the yellow and green colonies identified by colonial morphology and cultural characteristics of the colony as described by Pfeffer and Oliver (2003) and Kriem et al., (2015) [32, 33]. A single colony of presumptive isolates was then subcultured onto nutrient agar to ascertain purity, and each pure culture was picked and stored in glycerol stock for further analysis.

### Molecular confirmation of presumptive Vibrio species

The glycerol stocks were resuscitated using nutrient broth (Merck, Modderfontein, South Africa) and incubated for 24 h at 37 °C, while the genomic DNA of the 981 presumptive *Vibrio* spp., isolates were extracted following the boiling procedure described by [2, 34] with slight modifications. The fresh overnight bacterial isolates were sub-cultured into sterile 1.5 mL microfuge tubes and centrifuged (HERMLE, Siemensstr-25, D-78564 Wehingen, Germany) at a speed of 13,000 rpm for 10 min. The cell pellets were washed twice with phosphate-buffered saline, resuspended in 500 µL sterile distilled water, and then lysed to release the DNA by boiling at 100 °C for 10 min in pre-heated heating blocks (Techne heating block Dri-Block, DB-3D; Gauteng, Pretoria, South Africa). Afterward, the suspensions were centrifuged for 5 min at 15,000 rpm, and the supernatant was carefully pipetted into sterile Cryon tubes (Labotec, South Africa) and stored at − 20 °C.

The primer pair F-5′CGG TGA AAT GCG TAG AGA T-3′ and R-5′TTA CTA GCG ATT CCG AGT TC-3′ previously described by [35], was purchased from Inqaba Biotechnical Industries (Pty) Ltd., Pretoria, South Africa and used to amplify 16S rRNA genes of *Vibrio* spp., generating an amplicon size of ~ 663. The PCR reaction mixture of 25 µL (12 µL PCR master mix (New England BIOLABS), 1 µL of each forward and reverse primers, 6 µL of PCR grade water, and 5 µL of genomic DNA template were amplified using BioRad T100 thermal Cycler Lasec. (621BR44012, Singapore). Afterwards, 4 µL of the amplicons were electrophoresed in 1.5% agarose gel using the thermal tank (Labnet, Enduro Gel XL, USA) on staining with ethidium bromide (0.5 µL) and 0.5X Tris–borate EDTA (TBE) buffer with a controlled base size of 100-bp DNA ladder (New England BIOLABS), Madison, WI, USA). A 100 V and 60 min electrophoresis process was done, and the gels were visualized under the UV trans-illuminator (Alliance 4.7, UVItec, Merton, London, UK.

### Determination of virulence genes signature of the confirmed Vibrio species

The virulence gene signature distributions in the confirmed *Vibrio* spp isolates were determined using the PCR technique as we have described before [2, 36], with slight modifications. The set of primers indicates the targeted genes, sequence, and conditions in Table 1. The PCR reaction mixture was made up to a final volume of 25 μL, while the electrophoresed amplified amplicons were visualized as stated earlier.

**Table 1:** Primer pairs for traditional PCR screening and nucleotide sequencing of the virulence and resistance genes of the *Vibrio* species

Primer identity	Primer sequence	Amplicon length (basepair)	Reference
*strA*	FCTTGGTGATAACGGCAATTC	348	[37]
	R: CCAATCGCAGATAGAAGGC		
*aadA*	F: GTGGATGGCGGCCTGAAGCC	525	[38]
	R: AATGCCCAGTCGGCAGCG		
*aac(3)-IIa*	F: CGGAAGGCAATAACGGAG	428	[38]
*(aacC2)a*	R: TCGAACAGGTAGCACTGAG		
*sul1*	F: TTCGGCATTCTGAATCTCAC	625	[14]
	R: ATGATCTAACCCTCGGTCTC		
*sul11*	F: CGGCATCGTCAACATAACC		
	R: GTGTGCGGATGAAGTCAG		
*vcgCP1*	F: AGCTGCCGATAGCGATCT	278	[39]
	R: CGCTTAGGATGATCGGTG		

### Antibiotic resistance determinants using simplex PCR

The simplex PCR was used to assay relevant resistance determinants for the isolates obtained from phenotypic antibiotic-resistant *Vibrio* spp., isolates based on the susceptibility patterns [9, 34]. The resistance genes for the classes and specific antibiotics were assayed for including those of aminoglycosides [Kanamycin, Nitrofurantoin (*strA, aadA, aac(3)-IIa (aacC2)a*)]; and sulfonamides [Trimethoprime-sulfamethoxazole (*sul11*)]. The primers targeting conserved regions of the specific genes, sequence, cycle procedures, and expected amplicon band sizes are indicated in Table 1. All the PCR and electrophoresis procedures were carried out as earlier described.

### Partial nucleotide sequencing of amplicons and sequence analysis

For sequencing of amplicon gene analyses, the positive PCR products/amplicons of high quality were selected for sequencing at Inqaba Biotechnical Industries (Pty) Ltd. (Hatfield 0028, South Africa) using the forward and reverse primers earlier used in PCR amplification [40]. The amplicons/PCR products were purified and sequenced with standard Sanger sequencing [41]. Sequenced DNA were cleaned and edited in Bio Edith 3.3.19.0 and chromas 2.6.6 software, then blasted and assembled using Geneious 2021.1 [42]. As a first step, the DNA sequences were run via the Basic Local Alignment Search Tool (BLAST) to ensure that all of the sequences were genuinely *Vibrio* spp., compared to other GenBank sequences. Bioedit software [43] was used for nucleotide sequence alignment, whereas ClustalW, implemented in Geneious 10.1.2 software, was used for amino acid alignment [42].

### Restriction enzymes length polymorphism (RFLP) using six different digestive enzymes

The consensus sequence generated from Bioedit was used to analyze for RFLP by exploring the New England Biolabs restriction enzymes tools for analyzing DNA sequences at the site: http://nc2.neb.com/NEBcutter2/. 6 custom digest restriction enzymes were used to cut the DNA sequences, and predict the respective enzymes’ gel banding patterns [44]. The number of banding patterns produced per sequence was then counted and recorded respectively.

## Results

### A. Multiple alignments of the V. vulnificus virulence gene and three different isolates of the resistance genes

The gene investigated includes; [(*aac(3)-IIa, (aacC2)a*] aminoglycoside N(3)-acetyltransferase III, [*strA*] aminoglycoside 3′-phosphotransferase and [*aadA*] aminoglycoside (3′′) (9) adenylyltransferase*,* both resistance genes of (kanamycin, nitrofurantoin) aminoglycosides, and [*sul 1* and *11*] dihydropteroate synthase type 1 and 11 resistance gene of (trimethoprim-sulfamethoxazole) sulfonamides versus NCBI reference bacteria.

**Fig. 1:**
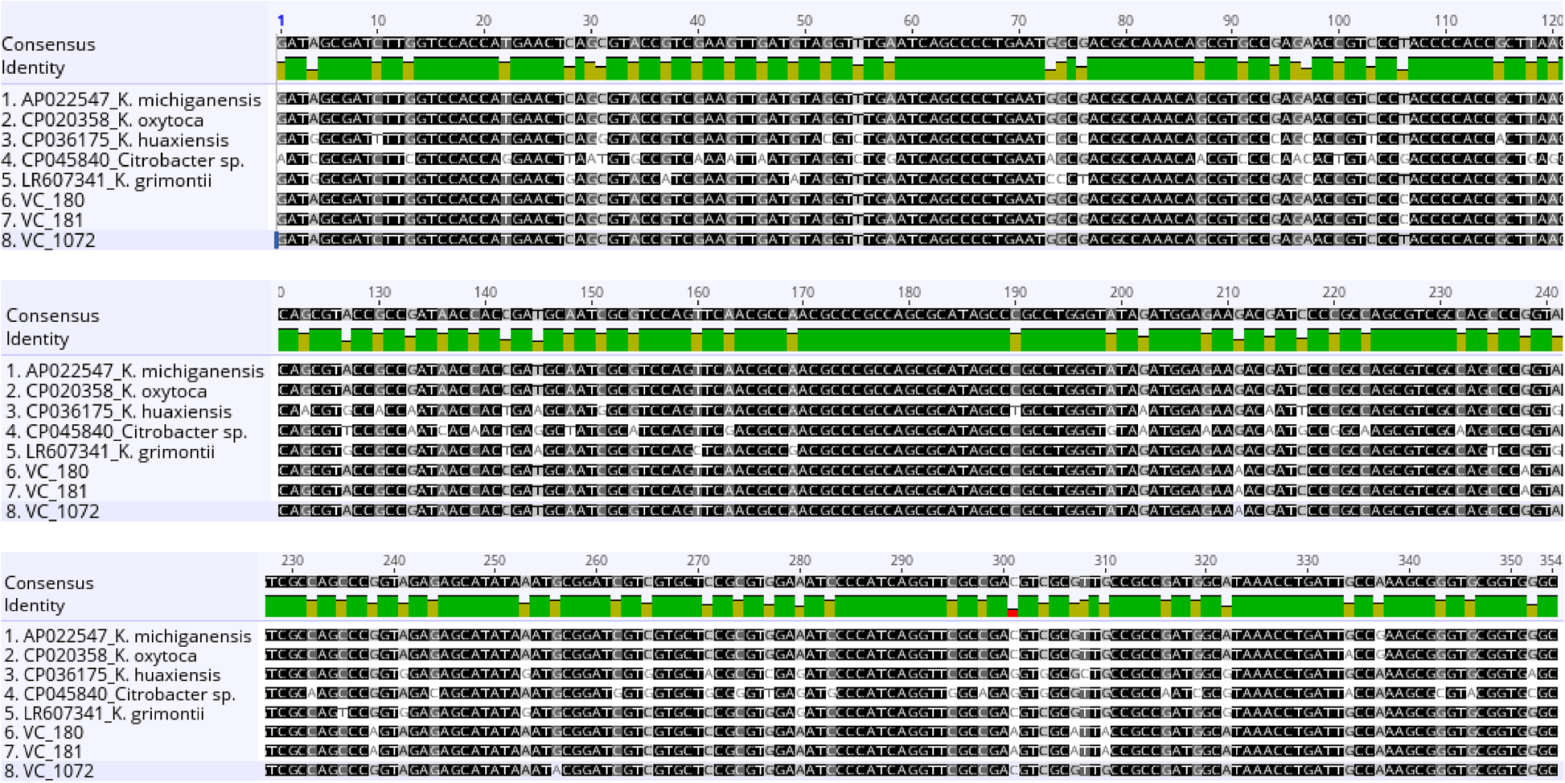
Nucleotide alignment of the partial genes *V. vulnificus* virulence gene (*vcgCPI*) obtained from Vibrio isolates with other reference bacterial species from the GenBank

**Fig. 2:**
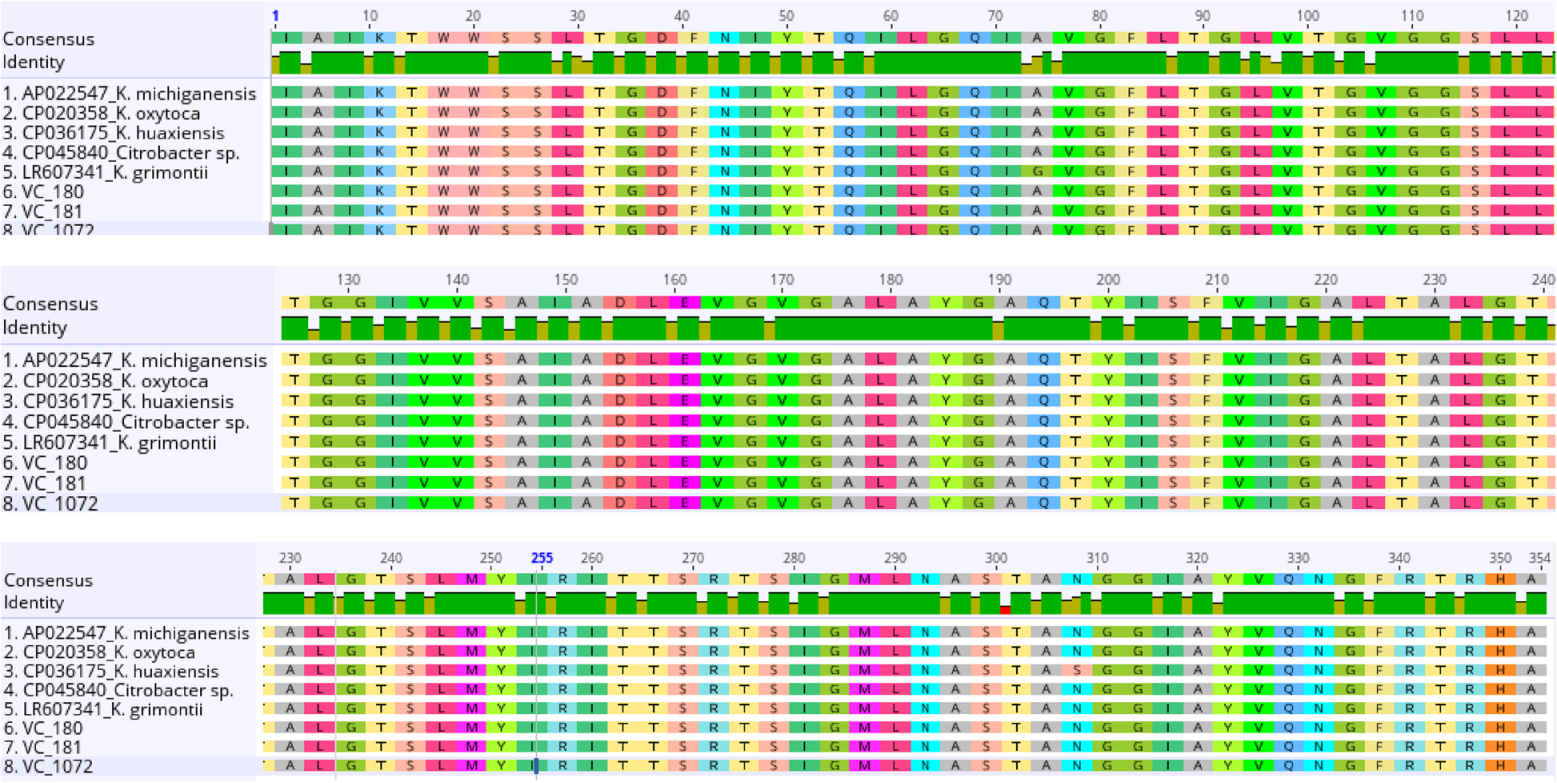
Putative amino acid sequences of the aligned V. *vulnificus* virulence gene (*vcgCPI*) as obtained in Geneious [42]

The multiple sequence alignment of the *V. vulnificus* virulence gene (*vcgCPI*) represented as (VC) genes obtained from *Vibrio* isolates in this study and other reference bacterial species show numerous nucleotide variations at different locations (Fig. 1). However, the nucleotide sequence polymorphism and mutation only result in similar putative amino acids in the virulence reference isolates, such as *K. grimontii* (LR607341) and *K. huaxiensis* (CP036175) at nucleotide positions 73–75 (A → G) and 300–302 (N → S) (Figs. 1 and 2).

**Fig. 3:**
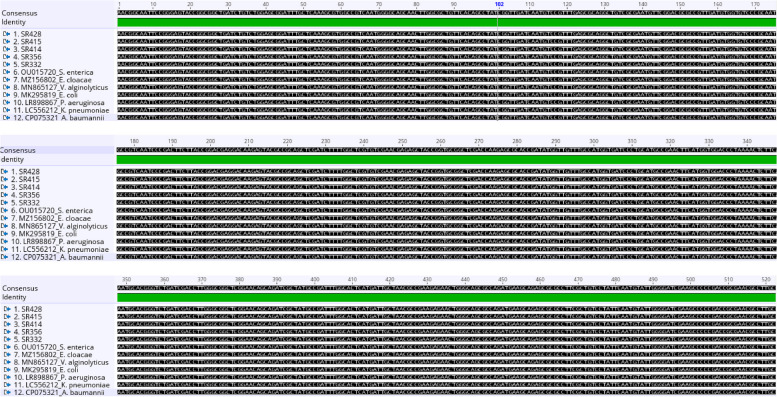
The multiple sequence alignment of partial (*aadA*) resistance genes

Figure 3 shows the nucleotide and amino acid sequence alignment of the five aminoglycosides resistance gene (*strA*) of *Vibrio* spp represented as (SR) obtained from *Vibrio* isolates in this study and seven other reference bacterial species. It could be deduced that the partial SR gene region of the *Vibrio* isolates sequenced is highly conserved; no single nucleotide difference was observed among the five sequences compared with all the reference bacterial species analyzed. Also, the putative amino acid sequences of the aligned SR are shown in the Supplementary file.

Nucleotide sequence alignment of partial five aminoglycosides resistance gene (*strA*) of *Vibrio* spp (SR) genes obtained from *Vibrio* isolates with sequences of different reference bacterial species from the GenBank.

**Fig. 4:**
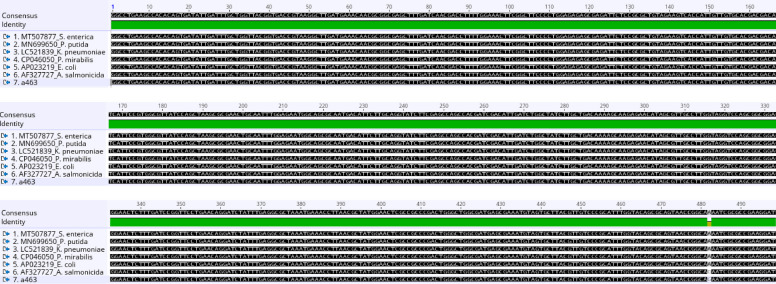
Nucleotide sequence alignment of the one partial (aadA) gene obtained from Vibrio isolates with six reference bacterial species

**Fig. 5:**
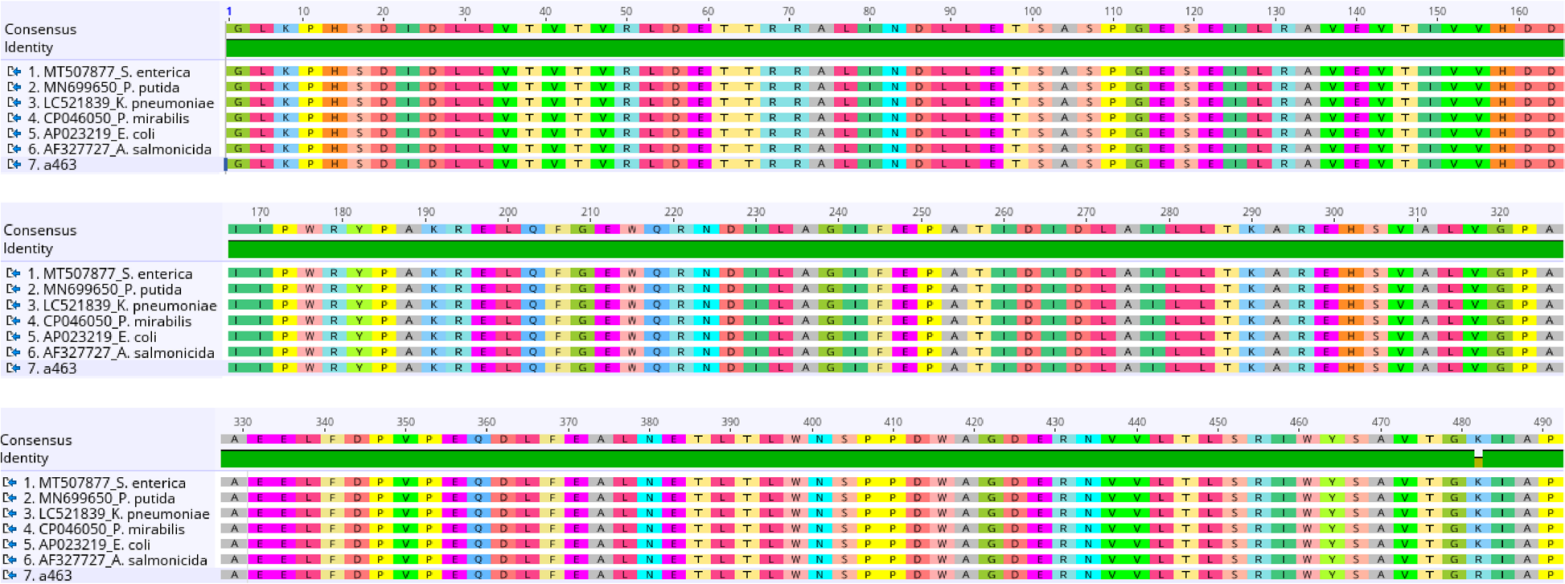
Deduced amino acid sequences of the aligned (aadA) genes. Multiple sequence alignment (MSA) of partial (*sul 1* and *11*) genes represent as (S) genes

The outcome of the nucleotide sequence alignment of the aminoglycosides resistance gene (*aadA*) of *Vibrio* spp., represented as (a) gene, from the *Vibrio* isolates in this study, with other six different reference bacteria species from the GenBank, equally showed high-level genome conservation across the different bacterial species, as only one nucleotide difference was observed at position 482 (A → G) for both *a463* and *Aeromonas salmonicida* (AF327727) (Fig. 4). However, the nucleotide difference does not vary in the amino acid sequence at the different bacterial species (Fig. 5).

**Fig. 6:**
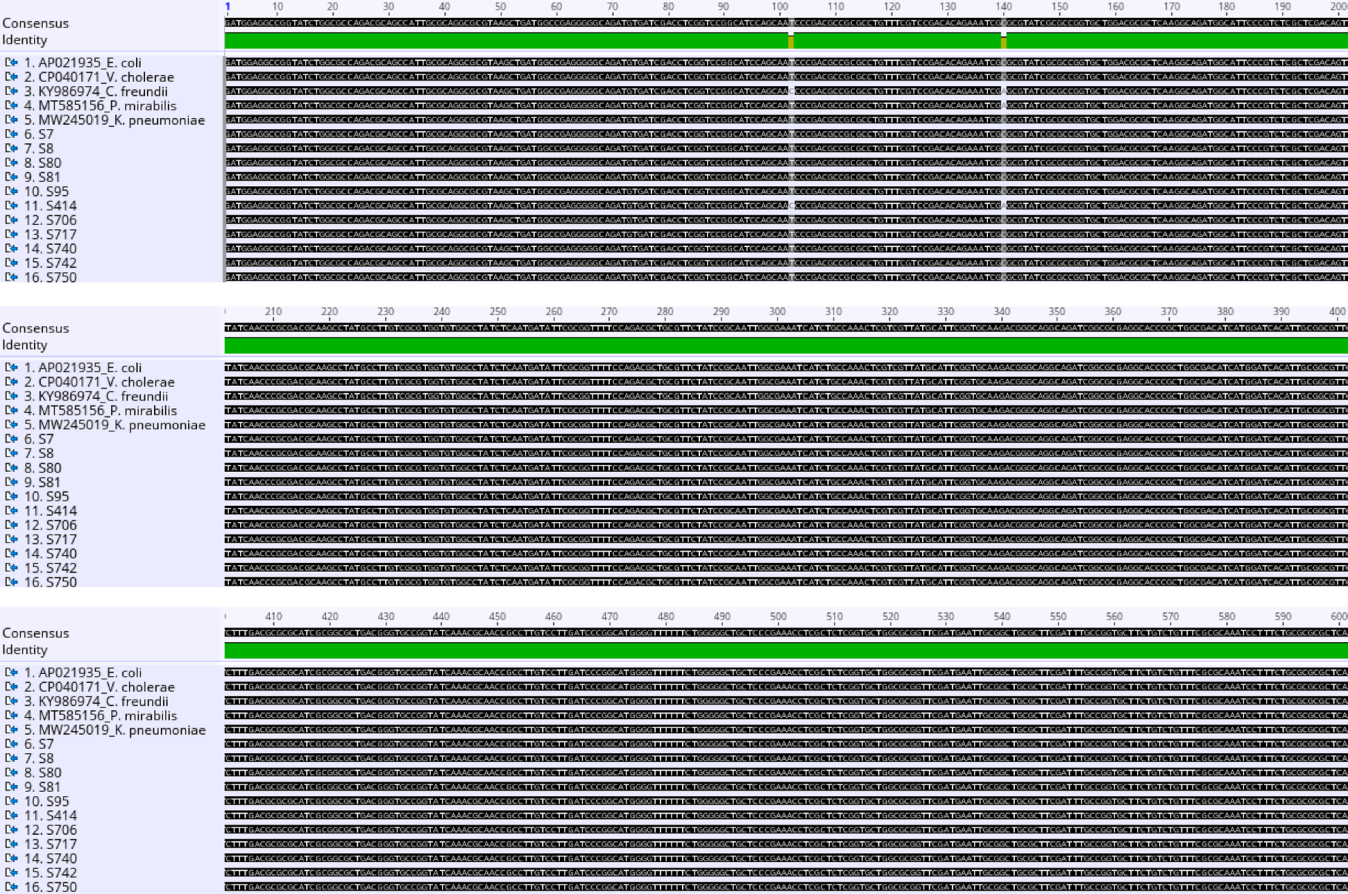
Nucleotide alignment of the 11 partial genomes obtained from (*sul 1* and *11*) (S) *Vibrio* isolates with five other reference bacterial species from the GenBank

**Fig. 7:**
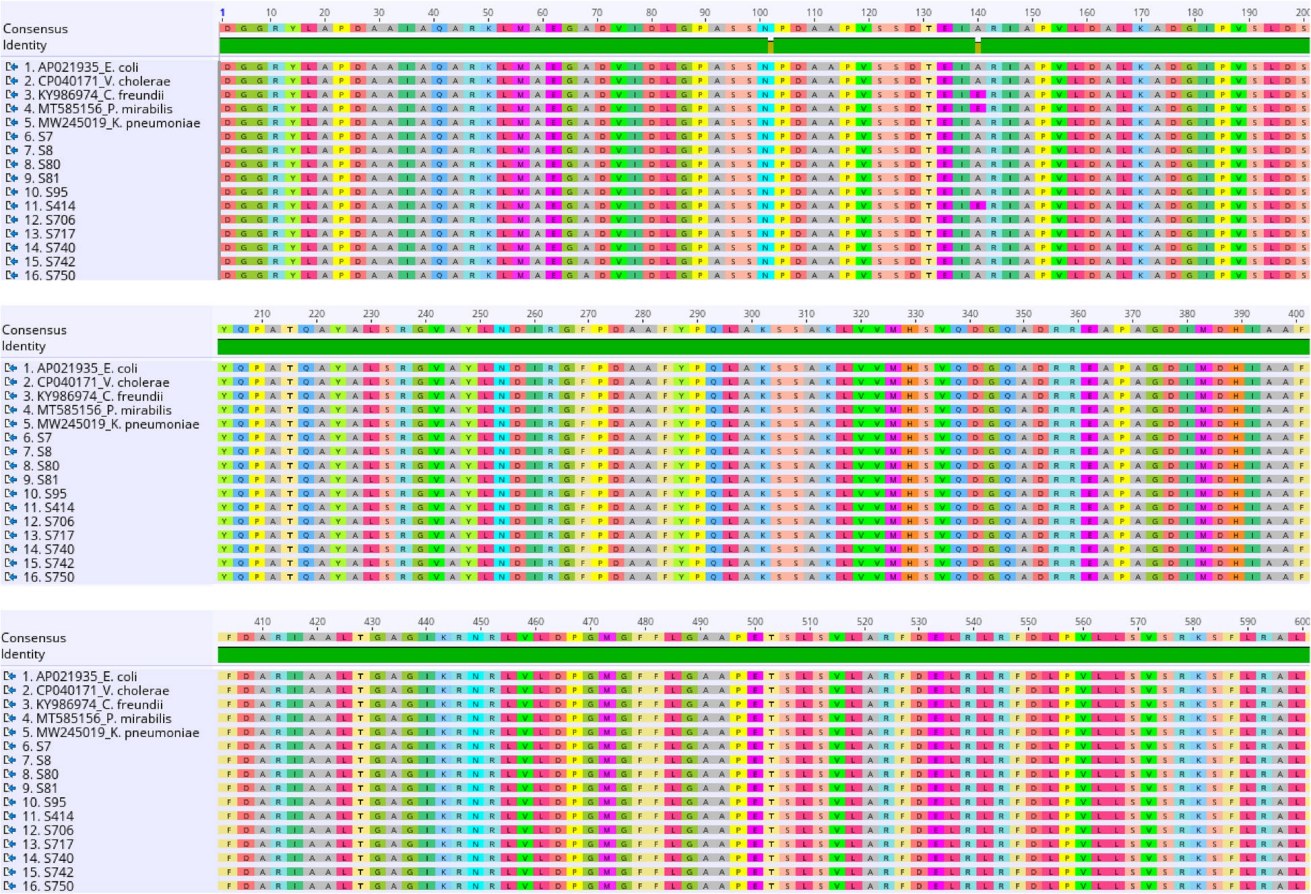
Putative amino acid sequences of the aligned (*sul 1* and *11*) (S) genes

The nucleotide sequence alignment of the 11 aminoglycosides resistance gene (*sul 1* and *11*) represented as (S) genes obtained from *Vibrio* isolates in this study and five different reference bacterial isolates from the GenBank, equally showed a high level of conservancy with only two observed regions of nucleotide polymorphism (102 and 140) as shown in Fig. 6. Sequence *S414* from this study has nucleotide ‘C’ at position 102 alongside the reference *C. freundii* (KY986974), while other reference bacterial species and the remaining ten sequences obtained in this study have ‘T’ at the same position. Also, at position 140, the sequence *S414* has ‘A’ together with the reference *C. freundii* (KY986974) and *P. mirabilis* (MT585156), while other sequences have nucleotide ‘C’ at the same position (Fig. 6). However, amino acid differences only exist due to the nucleotide polymorphism at position 140 (A → E), as shown in Fig. 7. The MSA was done in Geneious Prime 2021.0.3 [42]*.*

### B. Restriction enzymes length polymorphism using six different digestive enzymes

The result of the banding patterns as produced by the restriction enzymes show no significant differences among the *Vibrio* isolates and reference bacterial isolates extracted from the GenBank. While only the banding pattern produced in isolate *a3_966*, when digested by *Hinf1*, was significantly different with one band compared to the others and referenced bacterial isolates from the GenBank and when the restriction enzymes were combined as seen in A (MT151380 *V. cholerae*) (Table 2).

**Table 2:** Restriction enzymes length polymorphism analysis of [(*aac(3)-IIa, (aacC2)a*] using five different digestive enzymes on the Sequence resistance genes of 3 isolates of sequence analysis of (aac(3)-IIa, (aacC2)a; aminoglycoside N(3)-acetyltransferase III gene

Restriction enzymes	Isolates (sequence genes)			Positive control from NCBI		
	*a3_966*	*a3_969*	*a3_974*	A	B	C
*DpnI*	3	3	3	3	3	3
*EcoRV*	2	2	2	2	2	2
*Hinf1*	3	5	5	5	5	5
*HinP1I*	5	5	5	5	5	5
*NlaIV*	3	3	3	3	5	3
*Sac11*	2	2	2	2	2	2
CRE	6	7	7	5	7	7

*A* MT151380 *V. cholerae*; *B* CP047406 E. coli MS6; *C* CP054305 *K. pneumoni*; *CRE* combined restriction enzymes

The banding patterns produced by the restriction enzyme endonuclease digestion of virulence-correlated gene (*vcgCPI*) differed significantly. The isolate *VC_181* showed a higher banding pattern to the restriction enzymes, i.e., *HinP1I*, *MwoI*, and *StyD4I*, compared to others used and the reference bacterial isolates from the GenBank. In comparison, the banding patterns produced no significant differences when the isolates were digested with the enzymes *DpnI* and *Hinf1*. However, the reference bacteria CP071393 *K. Michigan*; CP036175_*K. huaxiensis* produced only a single band when digested with the *StyD4I* restriction enzyme in Table 3.

**Table 3:** Restriction enzymes length polymorphism analysis of (vcgCPI) using five different digestive enzymes on the three isolates of a virulence-correlated gene (vcgCPI)

Restriction enzymes	Isolates (sequence genes)			Positive control from NCBI	
	*VC_180*	*VC_181*	*VC_1072*	A	B
*DpnI*	4	4	4	4	4
*Hinf1*	2	2	2	2	3
*HinP1I*	3	4	3	2	2
*MwoI*	4	5	4	4	5
*StyD4I*	3	2	3	1	1
Combined restriction	6	4	6	6	6

*A* CP071393 *K. michigan*; *B* CP036175_*K. huaxiensis*

The banding patterns produced by the restriction enzyme digestion of protein dihydropteroate synthase type 1 and type II genes (*Sul 1* and *11*) differed significantly. The isolate *S_406* showed no band when digested with the restriction enzyme *NlaIV* compared to others and the reference bacterial isolates from the GenBank. Generally, variably different banding patterns were observed among all the isolates when digested with the enzymes *DpnI*, *HinP1I*, *NlaIV*, *MwoI*, and *StyD4I,* as shown in Table 4.

**Table 4:** Restriction enzymes length polymorphism analysis of *Sul 1* and *11* using five different digestive enzymes on the sequence resistance genes of 12 isolates of protein dihydropteroate synthase type 1 and type II genes (*Sul 1* and *11*)

RE	Isolates (sequence genes)												PC NCBI	
	*S_7*	*S_8*	*S_80*	*S_81*	*S_95*	*S_406*	*S_414*	*S_706*	*S_717*	*S_740*	*S_742*	*S_750*	A	B
*DpnI*	4	5	6	5	5	6	5	5	6	5	5	5	4	4
*HinP1I*	5	8	7	7	7	5	7	7	8	8	5	7	6	6
*NlaIV*	4	5	3	4	4	0	5	5	5	4	4	4	4	4
*MwoI*	7	8	7	6	7	4	8	7	6	6	7	6	6	6
*StyD4I*	4	3	3	3	3	2	3	3	5	5	3	3	3	3
CRE	3	5	5	4	4	5	4	2	3	4	4	3	4	4

*RE* restriction enzymes; *CRE* combined RE; *PC* positive control from NCBI

## Discussion

Since the endemic of *Vibrio* spp, phenotype variation is frequently used to determine or measure pathogenicity, intraspecies diversity by utilizing metabolizable substrates [45], colony morphotype [46], the presence of membrane proteins and lipopolysaccharide [47], extracellular enzymes such as cytolysins [48, 49], siderophores [50], virulence in mice [51], and resistance to animal host defense systems [52, 53], genetic divergence remain a prompt strategy for virulence determination. The preliminary phenotypic only provides appreciated evidence about the incidence and occurrence of phenotypic identities among *V.* strains. However, there is still a dearth of information on the characteristics of species mutation in order to predict strain pathogenicity and antibiotic treatment efficacy accurately.

The nucleotide and amino acid alignment results depict a diversity of alterations and mutations in the *V. vulnificus* virulence (*vcgCPI*) gene. Among the alterations, only the mutation at codons 309 nucleotide bases significantly affects the protein function of S (serine) compared to others. However, epidemiological studies have implicated the *vcgC* in clinical *Vibrio* isolates while the *vcgE* documented in environment isolates [39, 54]. The nucleotide polymorphisms observed within the genetic loci *vcg* allele show an incomparable likeness to the genetic characteristics frequently found in environmental isolates, as previously reported by D’souza et al. 2020) [55]. The nucleotides and amino acid alignment of [*strA*] Aminoglycoside 3′-phosphotransferase show no mutation or alteration in the gene sequences, possibly due to the highly conversed regions of the targeted gene.

The gene [*aadA*] Aminoglycoside (3′′) (9) adenylyltransferase shows a significant mutation at codon 482, which indicates a change in protein function of (Lys/K) Lysine found in the reference bacteria to (Arg/R) Arginine in the isolate a463. This observation may play a complementary protagonist in advancing high levels of aminoglycosides (e.g., Kanamycin and Nitrofurantoin) resistance, similar to the report shown by Minarini and Darini (2012) quinolone and ciprofloxacin resistance. Similarly, a significant mutation was observed in (*sul 1* and *11*) Dihydropteroate synthase type 1 and 11 genes at codons 102 and 140 of the isolate *S_414*. This alteration in codon 102 (T-C) was insignificant, as no implication was found in the putative amino acid. Nevertheless, the alteration at codon 140 (C–A) significantly affects the protein function causing a mutation of (Pro/P) Proline to (His/H) Histidine. This result is similar to the previous findings by Weigel and colleagues, which suggested that a substitution or mutation in an amino acid is sufficient to generate a significant degree of resistance to antibiotics, such as mutations in (Ser-83) for nalidixic acid resistance and in Thr83-Ile resistance to fluoroquinolones [56], alteration in Thr83-Ile resistance to ciprofloxacin [57].

The application of the RFLP technique to determine genomic relatedness of virulence or resistance genes and determine polymorphism among isolated *Vibrio* spp at various loci have been previously documented, e.g., [58, 59]. The results of restriction enzyme digestions by *DpnI*, *EcoRV*, *Hinf1*, *HinP1I*, *NlaIV*, and *Sac11* as well as in combinations as utilized in this study revealed that the majority of the *Vibrio* strains and the reference strains examined share similarity among the selected endonucleases. Specifically, the gene (*aac(3)-IIa*, (*aacC2)a* showed a difference of two bands when digested by hint restriction enzymes. This homology may be due to spontaneous translucent isolate, as previously observed by [51, 60]. Most *Vibrio* strains that have been previously reported are translucent strains, which are different from their opaque parent in the number of capsules produced. Therefore, the results from this study may be very likely due to the differences in physiological characteristics exhibited by these recent isolates. The different banding patterns observed in this study imply that these isolates possess unique pathogenic/biochemical characteristic polymorphism. As the different restriction enzymes (i.e., *DpnI*, *EcoRV*, *Hinf1*, *HinP1I*, *NlaIV*, *Sac11*) tested with nucleotide sequences from three isolates with the same (aac(3)-IIa, (aacC2) an Aminoglycoside N(3)-acetyltransferase III gene, the enzymes (*DpnI*, *HinP1I*, *NlaIV*, *MwoI*, *StyD4I*) tested on several isolates protein dihydropteroate synthase type 1 and type II (*Sul 1* and *11*) genes, and *vcgCPI* of which the endonuclease(s) produced polymorphic locus of DNA fragments.

Pulsed-field gel electrophoresis (PFGE) has been effectively used to discriminate strains of *Vibrio* as a powerful tool for differentiating bacterial strains [58, 61–63] after genomic cleavage of site-specific, low-frequency restriction endonucleases, exploiting the basic principle of movement of large DNA fragments in gels. This approach is extensively applied in epidemiological studies and less relatively employed in environmental investigation. Therefore, the observed polymorphism among RFLP profiles for tested genes in these environmental *Vibrio* strains indicates a polymorphic pathogenic/virulence relevance and treatment/management pattern.

## Conclusion

In conclusion, the isolates recovered from the surface water in greater Bushenyi encompass a very diverse population of *Vibrio* spp strains, and those specific subclasses of strains are pathogens that appear to be linked with human disease. The described virulence and resistance determinants possess specific polymorphic locus that may be relevant in pathogenomics, pharmacogenomics, vaccine production, and the control of strains in the future. Ultimately, this approach can provide scientific and rational bases for risk assessment.

### Supplementary Information

**Additional file 1: Supplementary file 1.** The putative amino acid sequences of the aligned SR

## Data Availability

The datasets/information used for this study are available from the corresponding author on reasonable request.
