# Supplementary material for: Polymorphism and mutational diversity of virulence (vcgCPI/vcgCPE) and resistance determinants (aac(3)-IIa, (aacC2, strA, Sul 1, and 11) among human pathogenic Vibrio species recovered from surface waters in South-Western districts of Uganda
Source: J Genet Eng Biotechnol. 2023 Oct 6;21:94. doi: 10.1186/s43141-023-00554-1 (PMC10558413; doi:10.1186/s43141-023-00554-1)
Supplement: Supplementary file 1 — Additional file 1: Supplementary file 1. The putative amino acid sequences of the aligned SR [file 43141_2023_554_MOESM1_ESM.docx]

**Supplementary Materials:** The putative amino acid sequences of the aligned SR are shown in Supplementary Figure 4.


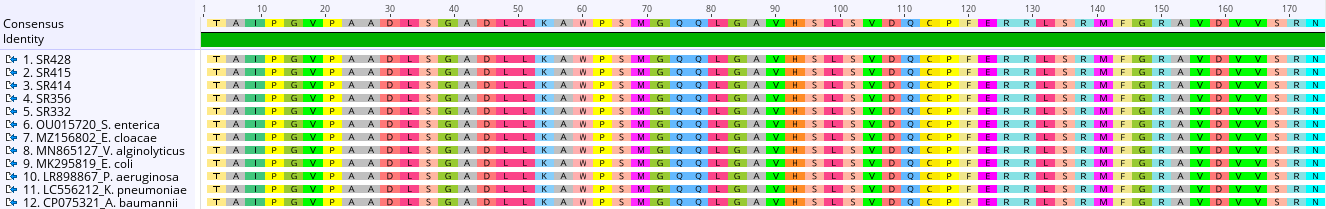


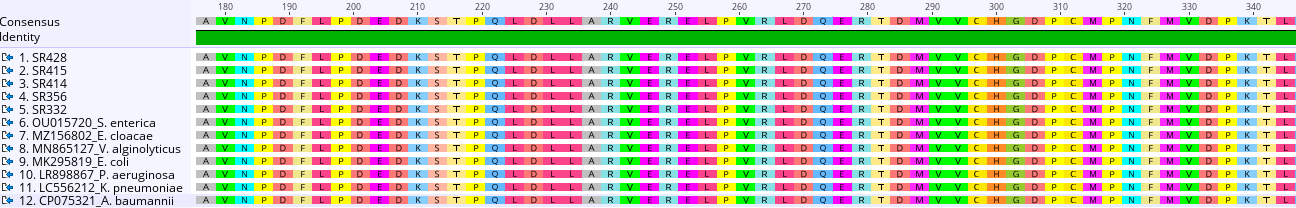


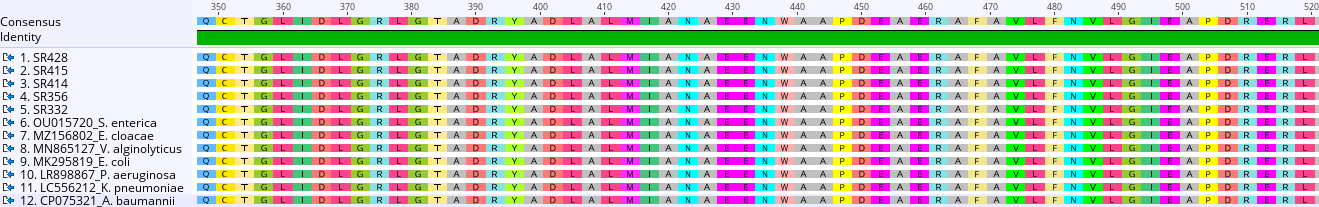


Figure 4.
